# Achieving a 3D Thermally Conductive while Electrically Insulating Network in Polybenzoxazine with a Novel Hybrid Filler Composed of Boron Nitride and Carbon Nanotubes

**DOI:** 10.3390/polym12102331

**Published:** 2020-10-13

**Authors:** Yi Wang, Wei Wu, Dietmar Drummer, Chao Liu, Florian Tomiak, Kevin Schneider, Zhengqiang Huang

**Affiliations:** 1 Sino-German Joint Research Centre of Advanced Materials, School of Materials Science and Engineering, East China University of Science and Technology, Shanghai 200237, China; Y20150057@ecust.mail.edu.cn (Y.W.); Y30170440@ecust.mail.edu.cn (C.L.); Y30180265@ecust.mail.edu.cn (Z.H.); 2 Institute of Polymer Technology, Friedrich Alexander University Erlangen-Nuremberg, 91058 Erlangen, Germany; drummer@lkt.uni-erlangen.de (D.D.); tomiak@lkt.uni-erlangen.de (F.T.); schneider@lkt.uni-erlangen.de (K.S.)

**Keywords:** fillers, functionalization of polymers, networks, nanocomposites

## Abstract

To solve the problem of excessive heat accumulation in the electronic packaging field, a novel series of hybrid filler (BN@CNT) with a hierarchical “line-plane” structure was assembled via a condensation reaction between functional boron nitride(f-BN) and acid treated carbon nanotubes (a-CNTs). The reactions with different mass ratios of BN and CNTs and the effect of the obtained hybrid filler on the composites’ thermal conductivity were studied. According to the results, BN@15CNT exhibited better effects on promoting thermal conductivity of polybenzoxazine(PBz) composites which were prepared via ball milling and hot compression. The thermally conductive coefficient value of PBz composites, which were loaded with 25 wt% of BN@15CNT hybrid fillers, reached 0.794 W· m^−1^· K^−1^. The coefficient value was improved to 0.865 W· m^−1^· K^−1^ with 15 wt% of BN@15CNT and 10 wt% of BN. Although CNTs were adopted, the PBz composites maintained insulation. Dielectric properties and thermal stability of the composites were also studied. In addition, different thermal conduction models were used to manifest the mechanism of BN@CNT hybrid fillers in enhancing thermal conductivity of PBz composites.

## 1. Introduction

Electronic and telecommunication industries have constantly developed for over half a century, corresponding to Moore’s Law [1]. However, this development seems to have slowed down in recent years. If we evaluate the progress of computers with the computing power per joule for the last 50 years, it can be observed that the ability of computers doubled every 2.5 years, which is a little slower than that predicted by Moore. The main reason is the loop of heat accumulation- increasing operating temperature- extensive heat generation. Part of the input energy is transferred into heat during operation, which accumulates in the electronic components and causes increasing working temperature. Then more energy is transferred into heat owing to the rising temperature. The growing packing density accelerates this process because of the increasing power density [2]. A report revealed that every 2 degrees increase in temperature would lead to a 10% degradation in the performance of electronic devices [3].Therefore, developing efficient heat-dissipating materials is a critical task for preserving reliability [4].

Polymers including polyamide (PA6), epoxy, etc. [5], have been used as packaging materials because of their low density, low cost and high processability [6]. A newly developed thermosetting phenolic resin polybenzoxazine (PBz) [7], is a promising material for the usage in microelectronics and packaging industries [8]. Polybenzoxazine, which is synthesized through Mannich condensation from amine, formaldehyde and phenol [9], possesses excellent properties such as low moisture absorption, stable dielectric constant and flame retardance [10]. In addition, PBz has splendid mechanical properties and thermal properties [11].

However, their application has been limited because of the relatively low intrinsic thermally conductive coefficient (λ) [12] (0.1–0.3 W· m^−1^· K^−1^). The most widely used approach is to simply blend polymers with fillers with high λ values, including carbon-based materials, metal, and ceramic [13,14]. In general, with the same content of filler, λ values of the composites filled with carbon-based fillers, such as graphene or carbon nanotubes (CNTs) [15], are more likely to be higher than those of the composites filled with ceramic fillers such as boron nitride. CNTs(~1300 W· m^−1^· K^−1^) [16] have been frequently used in fabrication of thermally conductive polymer composites, but the reported experimental results for the λ values of those composites are far less than those estimated from the rule of the simple blending model and the intrinsic thermal conductivity of CNTs [17]. Theoretically speaking, it is believed that the phonon scattering phenomenon in composite materials, achieved by acoustic mismatch, is the main reason impeding the further improvement of the thermal conductivity of those composites [18]. In addition, research revealed that when dispersed in polymers, CNTs are inclined to aggregate into bundles or ropes because of the strong intrinsic Van der Waals forces, which would also cause the diversity in theoretical and experimental results [19].

However, for applications in the electronics packaging field, polymer composites must be electrically insulating. Therefore, ceramic fillers have been widely employed, such as AlN [20], BN [21], SiC [22], etc. Among them, BN seems to be the most promising filler, owing to its high λ value (33 W· m^−1^· K^−1^~600 W· m^−1^· K^−1^) and relatively low dielectric constant (approximately four), compared with those of other ceramic fillers [23]. Lei, Y. [24] conducted research on the thermal conductivity of h-BN filled epoxy/cyanate resin composites. According to Agari’s model [25], the obtained data revealed that it is hard for h-BN to form effective conductivity channels. Therefore, fillers with large aspect ratios as well as high thermal conductivities [26], such as CNTs, were employed to assist the construction of the channels. Yang Xue [27] constructed a thermally conductive network with BN(30 phr) and CNTs (0.25 vol%) in silicone rubber, whose λ value was improved to 0.279 W· m^−1^· K^−1^, 25% higher than that without CNTs. This research revealed that CNTs contribute to the construction of thermally conductive channels. However, with simple blending, the agglomeration of CNTs can hardly be avoided, which means that only part of the CNTs contribute to connecting the BN platelets [28]. Moreover, because of CNTs’ interfacial boundaries and defect scattering, the agglomeration of CNTs undermines their ability to enhance thermal conductivity of the matrix [15].

Recently, controlling the distribution of filler to form robust heat transfer pathway has been proven to be an effective strategy to improve thermal conductivity of polymer-based composites [29]. For instance, Yimin Yao, et al. [30] adopted the combination of the ice-template and infiltrating method to construct BN platelets into a three-dimensional network, which were stacked on reduced graphene oxide(rGO). The reported 3D rGO-BN/epoxy composites exhibited an remarkable λ value of 5.05 W· m^−1^· K^−1^ at a filler loading of 13.16 vol.%. Yongqiang Guo et al. [2] constructed a new kind of fully carbon-based filler (f-MWCNT-g-rGO) with graphene oxide (GO) and multi-walled carbon nanotubes(MWCNTs). The thermally conductive polyimide nanocomposites (f-MWCNT-g-rGO/PI) were then prepared via a successive method of in situ polymerization, electrospinning and hot pressing. The fabricated fillers have a hierarchical structure, resulting in the outstanding thermal conductivity coefficient of 1.60 W· m^−1^· K^−1^ at a relatively low loading of fillers (10 wt%).

In this work, CNTs and h-BN were assembled together at different mass ratios via a condensation reaction to prepare a hybrid filler (BN@CNT) with a hierarchical “line-plane” structure, which was designed to effectively form a thermally conductive network throughout the polymer matrix. The BN@CNT/PBz composites were prepared via powder blending and then hot compression. The effects of structures of the as prepared hybrid fillers on thermal conductivity composites were investigated. In addition, PBz composites with high thermal conductivity were fabricated when BN and BN@CNT were simultaneously employed as fillers. Furthermore, to fully understand the effect of BN@CNT hybrid filler on enhancing thermal conductivity of PBz composites, the experimental data were fitted with a simple effective medium approximation (EMA) model and Foygel’s model to calculate thermal boundary resistance (R_B_) and contact thermal resistance (Rc).

## 2. Materials and Methods

### 2.1. Materials

Benzoxazine resin (BOZ) was supplied by Ruiyi Chemical Technology Co., Ltd.(Shanghai, China), (AIBZ682, density = 1.20 g· cm^−3^). Hexagonal boron nitride (50μm) (h-BN) was purchased from ZiboJonye Ceramic Technologies Co., Ltd. (Shandong, China).; carbon nanotubes (CNTs), with the average diameter of 30~50 nm and length of 10μm, were purchased from Shenzhen Zhongke Nano New Material Co. Ltd. (Shenzhen, China), Sodium hydroxide was supplied by Shanghai Aladdin Biochemical Technology Co., Ltd. (Shanghai, China); 3-triethoxysilylpropylamine (>98%) (APTES) was purchased from Shanghai Yuanye Biotechnology Co., Ltd.; N,N’-diisopropylcarbodiimide (DIC), sulfuric acid (98%), nitric acid (68%), absolute ethanol and tetrahydrofuran (THF) were all purchased from Shanghai Titan Scientific Co., Ltd. (Shanghai, China).

### 2.2. Specimen Preparation

a-CNT Preparation. To obtain the carboxyl grafted CNTs, 1 g CNTs was added into 30 mL sulfuric acid (98%) and 10 mL nitric acid (68%) and ultrasonicated for 4 h at 60 °C [31]. The slurry was centrifuged for 1.5 h, and then washed with DI water until the pH of the mixture was 6.5. After being ultrasonicated for 1 h, the slurry was placed in afreezer at −18 °C to be frozen for 12 h. After freeze-drying for 48 h, the acid-treated CNT (a-CNT) was obtained.

f-BN Preparation. Firstly, 20 g h-BN was dispersed in 200 mL 5 M aqueous sodium hydroxide solution and stirred magnetically for 24 h at 120 °C. The mixture was alternately filtered and washed three times with DI water to remove the redundant sodium hydroxide. After drying at 120 °C for 24 h, BN-OH was obtained. Secondly, APTES (3% of the weight of obtained BN-OH) was dissolved in a 95 wt% aqueous ethanol solution, the pH of which was adjusted to 5. Then BN-OH was added into the solution and stirred magnetically for 6 h at 80 °C. The slurry was then vacuum dried at 120 °C for 24 h. Finally, the obtained powder was washed three times with ethanol and DI water to remove unreacted APTES. After drying at 120 °C for 24 h, light yellow particles were obtained which were coded as f-BN.

**Figure 1:**
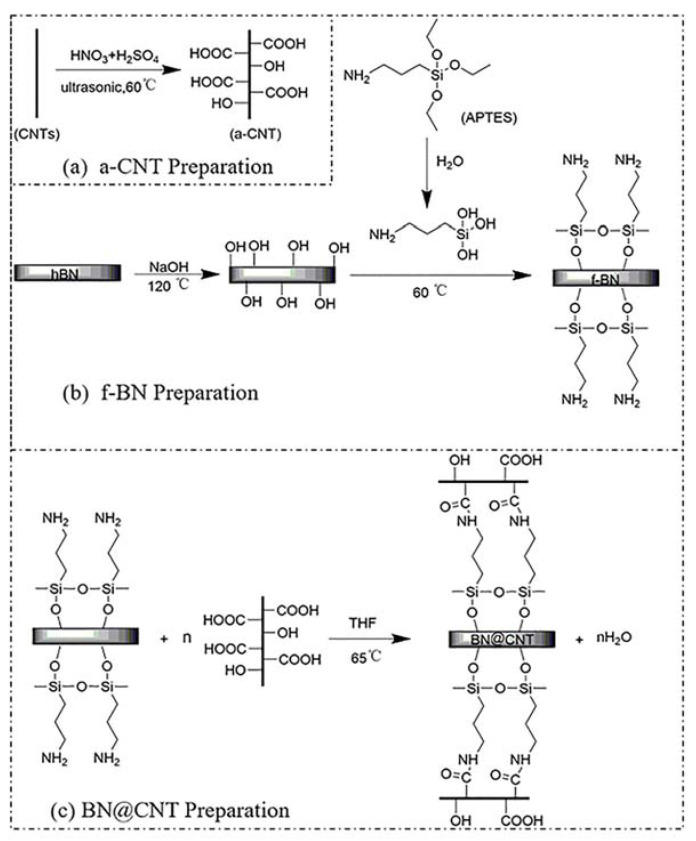
Illustration of the preparation procedures of BN@CNT hybrid filler.

BN@CNT Preparation. a-CNT, f-BN and DIC were added into THF, followed by ultrasonic treatment for 2 h and stirred magnetically for 48 h at 65 °C. The mixture was washed with DI water through centrifugation and underwent further drying. The formula is list in Table 1. The obtained particles were named as BN@xCNT(x = 5/10/15/20). The whole procedure is shown in Figure 1

**Figure 2:**
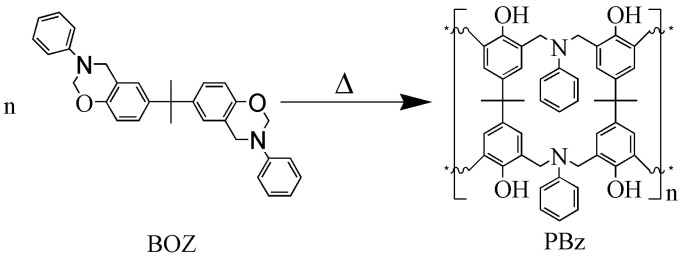
Curing mechanism of PBz.

Fabrication of thermally conductive BN@xCNT/PBz composites. As prepared BN@xCNT and BOZ were blended together through ball milling at 170 rpm for 2 h. Then the blended powder was filled into a certain mold and hot pressed under 12 MPa following the heating strategy of 160 ℃/1 h +180 ℃/1 h +200 ℃/1 h +220 ℃/1 h. The curing mechanism is shown in Figure 2. For comparison, BN/PBz composites, CNTs/PBz composites and BN/CNTs/PBz composites were prepared following the same strategy, in which BN/CNTs refersto simple blending of BN and CNTs at the same mass ratio as that of BN@15CNT.

### 2.3. Characterizations

Fourier transform infrared (FTIR) spectra was conducted on an IR spectrometer (IRAffinity-1, Kyoto, Japan) from 400 cm^−1^ to 4000 cm^−1^.

A scanning electron microscope (SEM, S-3400, Hitachi Ltd., Japan) was used to observe the morphology of the hybrid filler particle and fabricated composites. Samples were fractured in liquid nitrogen.

Thermogravimetric analysis (TGA) of the specimens were carried out by a 2P-WRT (Shanpin Instrument Co., Shanghai, Ltd., Shanghai, China) over a range of temperature (50–750 °C); all the filler samples were tested in air atmosphere at 10 °C/min, while polymeric composites were tested in nitrogen atmosphere at 10 °C/min.

A broadband dielectric spectrometer (Novocontrol Technology Company, Germany) was used to measure the dielectric constant (ε) and dielectric loss factor (tanδ) values of composites. The testing frequency range was from 10^−1^ to 10^7^ Hz. Samples were cylindrical shaped of 21.0 mm in diameter and 1 mm in thickness.

A high resistivity meter, model LK2679A was used to measure the volume resistivity (R) of composites at 250V. The corresponding dimension of specimen was 80 mm×80 mm×1 mm.

A thermal conductivity instrument TC3000E (Xi’an Xiaxi Electronic Technology Co., Ltd., Xi’an, China) was employed to measure the thermally conductive coefficient (λ) of the samples. The corresponding dimensions of the specimens were 60 mm × 40 mm × 2 mm.

## 3. Results and Discussion

### 3.1. Characterization of BN@xCNT Hybrid Filler

**Figure 3:**
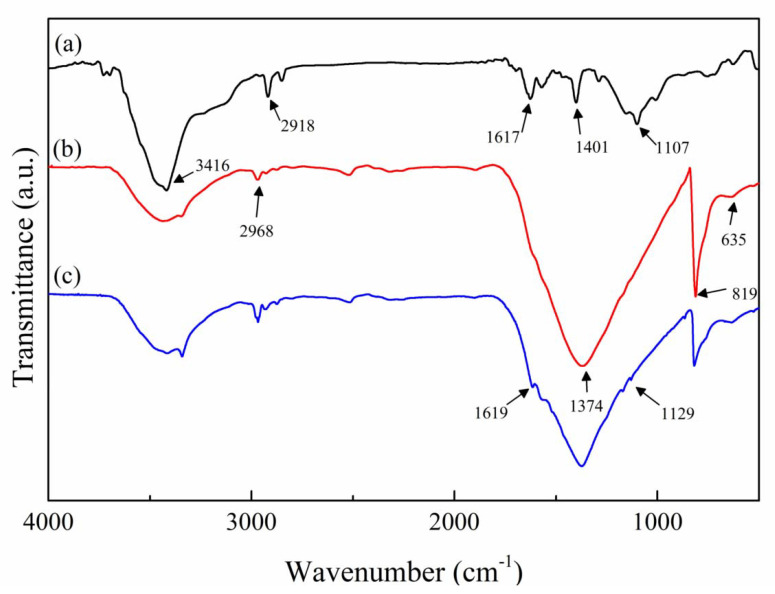
FT-IR spectra of (**a**) a-CNT; (**b**) f-BN; and (**c**) BN@15CNT.

As is shown in Figure 3a, peaks at 2918 cm^−1^ and 1401 cm^−1^ corresponded to the CNT core [32]. The peak at 3416 cm^−1^ resulted from the stretching vibration of –OH, and the stretching vibration of –C=O– and –C–O- appeared at 1617 cm^−1^ and 1107 cm^−1^, respectively. These peaks revealed that –OH and –COOH were introduced to CNTs after acid treatment. The characteristic absorption peaks of h-BN platelets can be seen in Figure 3b at 1374 cm^−1^ and 819 cm^−1^, indicating the B–N stretching vibration and the B–N–B out-of-plane bending, respectively [33]. Peaks at 2968 cm^−1^ (stretching vibration of C–H) and 635 cm^−1^ (out-of-plane bending vibration of N–H) indicated the existence of APTES. In Figure 3c, the peak at 1614 cm^−1^ resulting from stretching vibration of C=O revealed the formation of amide bonds, which connected f-BN and a-CNTs.

**Figure 4:**
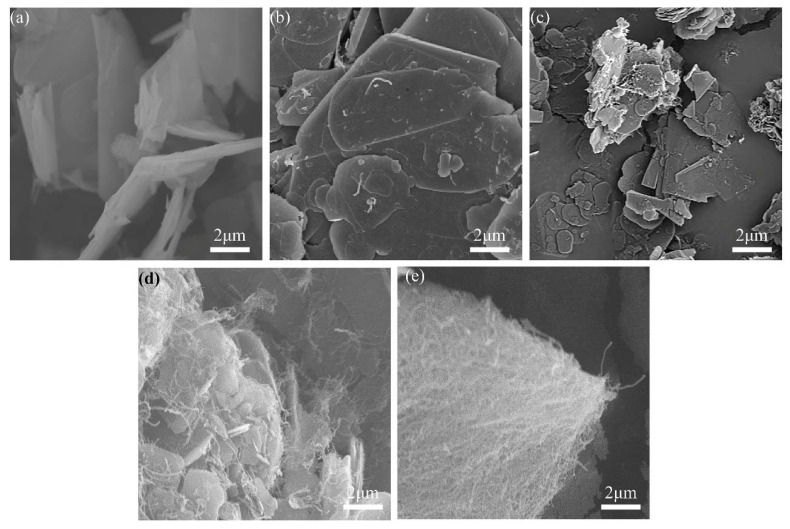
SEM image of BN@xCNT hybrid fillers of (**a**) pristine h-BN;(**b**) BN@5CNT; (**c**) BN@10CNT; (**d**) BN@15CNT; and (**e**) BN@20CNT.

Morphologies of BN@xCNT with different weight ratio of CNTs are shown in Figure 4. In Figure 4a the surface of BN flakes is smooth. With the increasing content of CNTs, the distribution of CNTs varied. In Figure 4b,c, it can be observed that most a-CNTs dispersed on the surface of h-BN with no sign of large agglomerate. With further increasing content of a-CNTs, it can be observed from Figure 4d that some of a-CNTs stacked at the edge of f-BN and connected several h-BN particles together. As seen in Figure 4e, a-CNTs covered the whole surface of f-BN when the content of CNTs kept increasing. It can be estimated that the differences in location of a-CNTs would lead to the differences in thermal conductivity of BN@xCNT/PBz composites.

**Figure 5:**
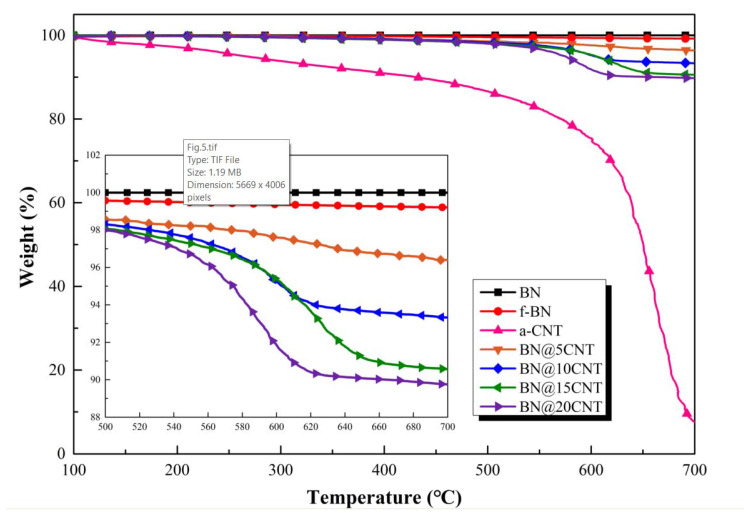
TGA curves of h-BN, f-BN, BN@xCNT and a-CNT.

To quantitatively analyze the content of CNT in prepared BN@xCNT, TGA under air atmosphere was conducted on pristine h-BN, f-BN, BN@xCNT and a-CNTs. The corresponding TGA curves are presented in Figure 5. The pristine h-BN platelet with high thermal stability did not show obvious weight loss. f-BN showed slight weight loss resulting from the decomposition of APTES [34]. Furthermore, a-CNT kept losing weight from 100 °C, resulting from the decomposition of –OH groups, –COOH groups and defect points formed during acid treatment. The obvious weight loss for a-CNTs over 550 °C was attributed to the decomposition of its core structure. BN@xCNT decomposed in a similar pattern as did a-CNT. According to following equations, the content of CNTs in prepared BN@xCNT and the conversion rate of the reaction can be calculated.

Φ = (l_1_ − l_2_)/(l_3_ − l_2_) (1)

α = Φ/Φ_0_ (2)

in which l_1_, l_2_, l_3_ are the weight loss percentage of BN@xCNT, f-BN and CNT, respectively; Φ_0_ and Φ stand for the content of a-CNTs before and after reaction; α means the conversion of the reaction. The data used and obtained results are listed in Table 2. It can be inferred from the α value that with the increasing of the a-CNTs and h-BN ratio, more a-CNTs were grafted onto f-BN, and a-CNTs were excessive when the ratio of CNTs and f-BN reached 1:5. Therefore, the α value of BN@20CNT dropped dramatically.

### 3.2. Effect of BN@xCNT Hybrid Fillers’ Structure on the Thermal Conductivity of PBz Composites

**Figure 6:**
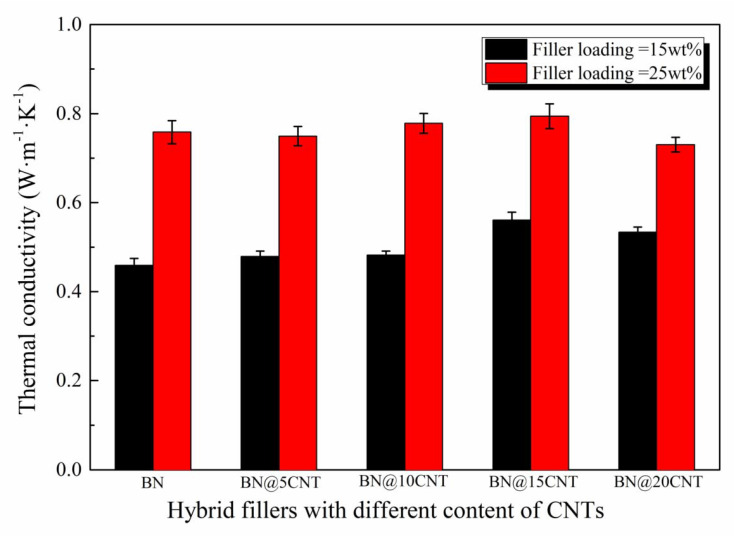
Thermal conductivity of PBz composites with 15 wt% and 25 wt% BN@xCNT hybrid fillers.

To fully determine the relationship between the content of CNTs in BN@xCNT hybrid fillers and their effect on thermal conductivity of corresponding PBz composites, filler loading over and below the percolation threshold were both investigated, since the pattern of thermal conductivity differs. According to former research, the percolation threshold in a similar system was about 20 wt% [35]. Therefore, 15 wt% and 25 wt% were chosen as the filler loading in this part. The corresponding λ values are shown in Figure 6.

**Figure 7:**
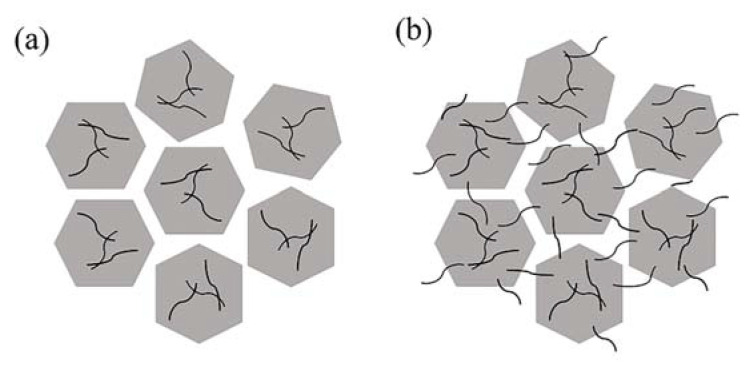
Schematic diagram of the effect of different BN@xCNT hybrid fillers on the heat transferring: (**a**) BN@5CNT; (**b**) BN@15CNT.

It can be observed that PBz composites filled with different BN@xCNT hybrid fillers performed various λ values at the same filler loading. Such a discrepancy was more obvious at lower filler loading. Among all the hybrid fillers, BN@15CNT showed the best ability to improving thermal conductivity of PBz composites than other fillers, both at 15 wt% and 25 wt% loading amounts. The corresponding λ values of PBz composites were improved from 0.459 W· m^−1^· K^−1^ and 0.762 W· m^−1^· K^−1^ to 0.564 W· m^−1^· K^−1^ and 0.794 W· m^−1^· K^−1^, respectively, comparing with pristine BN. It can be inferred that the differences in λ values were caused by the combined action between the structure of hybrid fillers and filler loading. When the content of filler was under the percolation threshold, the BN platelets were not connected with each other. Therefore, the construction of a thermally conductive network depended on the CNTs to connect adjacent BN platelets. When the filler loading was over the percolation threshold, BN platelets could form a thermally conductive network themselves, which means CNTs contributed less to the formation of the network. This explained why the discrepancy in λ values at 15 wt% were more obvious than that at 25 wt%. At the same filler loading, the different structure of hybrid fillers resulting from various CNT contents dominated the enhancement in λ values of the PBz composites. According to the SEM images of BN@5CNT and BN@10CNT hybrid fillers, the a-CNTs distributed on the surface of BN platelets so that they could hardly link the adjacent BN platelets, as shown in Figure 7a. Therefore, the corresponding λ values were almost similar to those of BN/PBz composites. When the a-CNT started to distribute on the edge of BN platelets, they could act as “bridges” to connect BN, which is shown in Figure 7b. In this way, the BN@15CNT hybrid filler exhibited the best ability to enhance the thermal conductivity of the PBz composites at both filler loading levels. It is worth noting that the λ value of BN@20CNT/PBz composites was higher than that of BN/PBz composites at 15 wt% filler loading while the opposite was the case at 25 wt% filler loading. This could be attributed to competition between serious phonon scattering of a-CNT and formation of a thermally conductive network [36]. In the BN@20CNT hybrid filler, a-CNT covered BN platelets thoroughly, so that when the hybrid fillers connected with each other, it was the connection between a-CNT in most cases. Due to lattice mismatch and defects caused by acid treatment, the phonon scattering between the a-CNT connection was serious and led to high contact resistance [37]. At 15 wt% filler loading, a-CNT assisted the formation of the thermally conductive network which did not exist in BN/PBz composites via connecting adjacent BN platelets. Therefore, BN@20CNT/PBz composites exhibited better thermal conductivity than BN/PBz composites did. However, at 25 wt% filler loading, BN could form a thermally conductive network even without a-CNT, so that phonon scattering became the main reason influencing thermal conductivity of BN@20CNT/PBz composites.

The following research focuses on the effect of the BN@15CNT hybrid filler on properties of PBz composites resulting from the fact that BN@15CNT exhibited the best ability to enhance thermal conductivity of PBz composites at both filler loadings compared with other fillers.

### 3.3. Effect of BN@15CNT on Thermal Conductivity of PBz Composites

**Figure 8:**
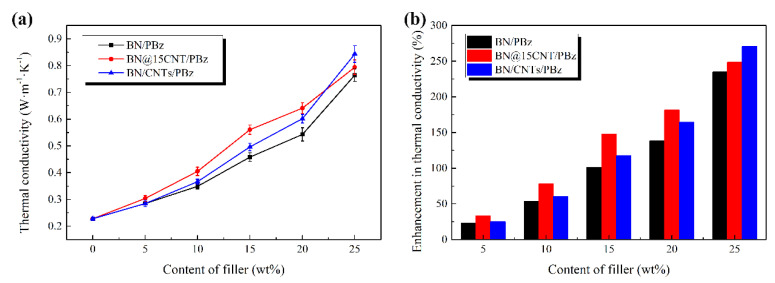
(**a**)Thermal conductivity of the PBz composites with different content of BN@15CNT and BN; (**b**) enhancement in thermal conductivity of BN@15CNT/PBz and BN/PBz composites.

Figure 8a depicts the λ values of BN@15CNT/PBz, BN/CNTs/PBz and BN/PBz composites. It can be observed that λ values of composites with the BN@15CNT or BN/CNTs were higher than those of composites with pristine BN at all filler loading levels. When the content of filler was no more than 20 wt%, the BN@15CNT hybrid filler exhibited a better effect on enhancing thermal conductivity of PBz composites than BN/CNTs did. However, when the filler loading reached 25 wt%, the λ value of BN/CNTs/PBz composite reached 0.844 W· m^−1^· K^−1^, while that of BN@15CNT/PBz composites and BN/PBz composites was 0.794 W· m^−1^· K^−1^ and 0.765 W· m^−1^· K^−1^, respectively. The corresponding enhancement reached 270%, 249% and 235%, respectively.

It can be observed from the λ values of PBz composites filled with these different fillers that the cooperation of BN and CNTs, either in physical or chemical methods, led to synergetic effects on enhancing thermal conductivity of PBz composites. The main reason is that CNTs served as “bridges”, establishing a connection between BN platelets, so as to improve the continuity of the thermal network. When the filler loading was less than 20 wt%, BN@15CNT/PBz composites showed better thermal conductivity than BN/CNTs/PBz composites did because the BN@15CNT hybrid filler ensured that CNTs distributed evenly among BN platelets so that the continuous thermally conductive network could be formed. However, in BN/CNTs/PBz composites, the distribution of CNTs would not be even because the simple blending of BN and CNTs could not avoid CNTs’ agglomeration, which led to the fact that part of the BN platelets were connected with CNTs while part of them lacked connection with other fillers. When the content of filler reached 25 wt%, BN platelets could form thermally conductive network themselves, so that the a-CNTs on their surface became less necessary while simple blended CNTs could form new channels because they were not constrained on the BN surface [38]. Moreover, the CNT connections between BN@15CNT hybrid fillers increased the contact resistance because of the phonon scattering caused by lattice mismatch when CNTs stacked together.

### 3.4. Further Enhancement in Thermal Conductivity via the Synergistic Effect Between BN@15CNT and BN

**Figure 9:**
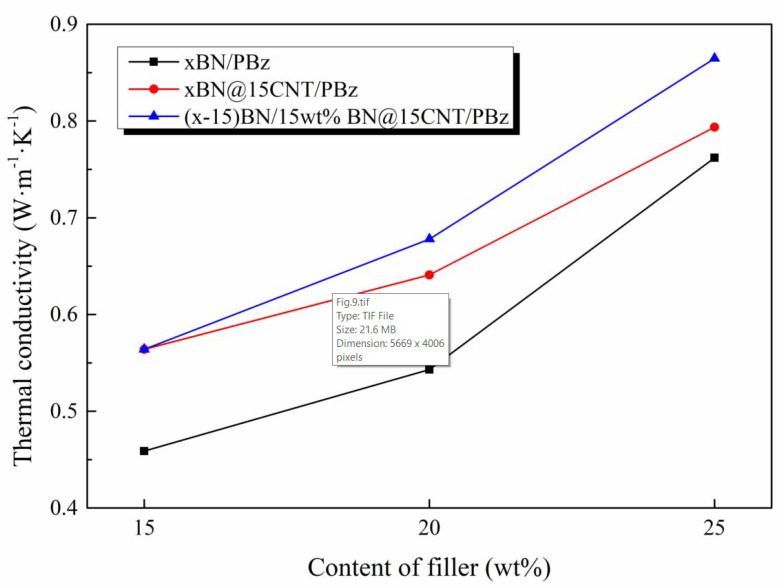
Thermal conductivity of the BN/BN@15CNT/PBz composites.

According to the former explanation, to achieve better enhancement in thermal conductivity, the interconnection between CNTs and BN should be maximized while that between CNTs should be avoided. To test this theory, BN was added into 15 wt%BN@15CNT/PBz composites. As it is shown in Figure 9, with the introduction of BN, the λ value of PBz composite increased more sharply than that of either BN@15CNT/PBz composites or BN/PBz composites. With 15 wt% of BN@15CNT and 10 wt% of BN, the PBz composite’s λ value reached 0.865 W· m^−1^· K^−1^. This obvious enhancement was ascribed to the fact that BN@15CNT offered sufficient “bridges” to connect other filler particles while BN dampened the chances of the overlapping of CNTs. When 15 wt% BN@15CNT hybrid filler was loaded, the thermally conductive network was about to be formed completely. Compared with further addition of BN@15CNT, the addition of BN would also help construct the network while the interconnection between CNTs would not increase, which conforms to the explanation proposed before.

### 3.5. Dielectric Properties of the BN@15CNT/PBz Composites

**Figure 10:**
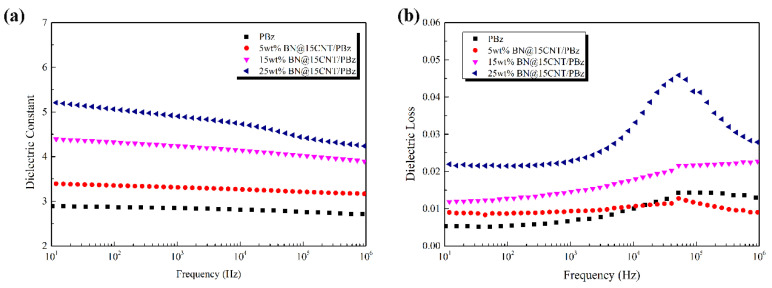
(**a**) ε and (**b**) tanδ values of PBz composites with different content of BN@15CNT at different testing frequency

The ε and tanδ values of the PBz composites with different content of BN@15CNT hybrid filler at different testing frequencies are presented in Figure 10. The ε and tanδ values both increased with increased filler loading at the same frequency. Except for the composite with 25 wt% filler content, ε values of the composites showed little dependency on frequency. However, the tanδ values firstly increased, then decreased, the tendency of which became more obvious with higher filler loading. This tendency indicated the process of dielectric relaxation [39]. The corresponding ε and tanδof the BN@15CNT/PBz composite with 25 wt% BN@15CNT hybrid fillers was 1.60 and 0.028 at 1 MHz, respectively.

It can be inferred from the peak in tanδ values that pristine PBz performed viscoelastic relaxation because of dipolar relaxation, in which these dipoles were permanent dipoles present on the side chains of the polymer backbone [40] When the filler loading reached 25 wt%, the relaxation behavior was extremely obvious, which resulted from the combination of viscoelastic relaxation and conductivity relaxation, which was due to translational diffusion of ions that caused conduction [41].

### 3.6. Electrical Conductivity of BN@15CNT/PBz Composites

**Figure 11:**
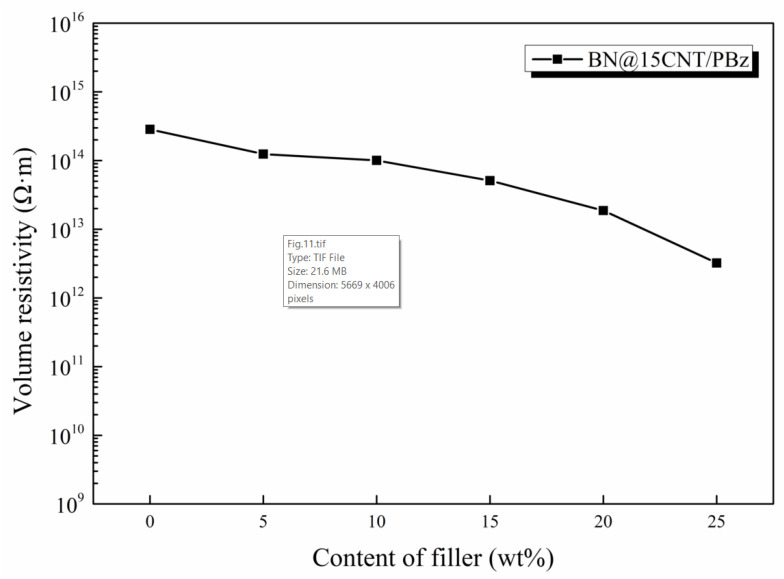
Volume resistivity of BN@15CNT/PBz composites as a function of filler content.

For the application in electronic packaging, composite materials must be electrically insulated (volume resistivity > 10^12^ Ω· m). Figure 11 shows the volume resistivity of the composites as a function of filler content. A decrease in volume resistivity of BN@15CNT/PBz composites could be observed with the increases in filler content. This can be explained by the microstructure of the hybrid fillers. For the BN@15CNT hybrid fillers, CNTs were restricted on the surface of h-BN platelets. Hence the low content of BN@15CNT hybrid fillers would not achieve enough contact of CNTs. Although the contact of CNTs would increase with the higher content of hybrid filler, the network of CNTs were separated by h-BN platelets, and therefore the high insulation property could be maintained.

### 3.7. Thermal Stability of BN@15CNT/PBz Composites

**Figure 12:**
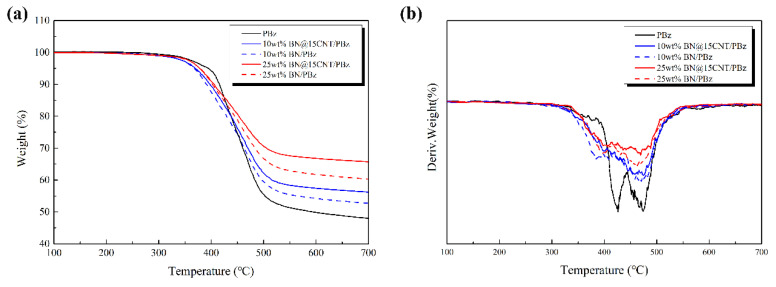
(**a**) TGA curves and (**b**) DTG curves of pristine PBz, BN/PBz composites and BN@15CNT/PBz composites.

To study the effect of BN@15CNT hybrid fillers on the thermal stability of PBz composites, BN@15CNT/PBz composites, BN/PBz composites and pristine PBz were characterized via TGA. The TG and DTG curves are shown in Figure 12 and corresponding characteristic thermal temperatures are listed in Table 3. It can be concluded that both BN@15CNT/PBz composites and BN/PBz composites exhibited a similar tendency, which is that the decomposition temperature of composites was reduced with the introduction of filler, although the higher content filler led to higher thermal decomposition temperature. The DTG curve of pristine PBz exhibited two peaks, revealing the two step decomposition of PBz, while only one peak could be observed in DTG curves of PBz composites. This tendency revealed that the introduction of fillers blocked the movement of molecules and reduced the crosslink density of the matrix, leading to the decrease in decomposition temperature of composites [42]. With further increases of filler contents, the degradation of PBz molecular chains was delayed because improved thermally conductivity of the composites led to the rapid spread of heat and ultrahigh specific heat capacity of BN resulted in the absorption of more heat.

In addition, the TG curves of BN@15CNT/PBz composites showed some interesting differences compared with that of BN/PBz composites while the DTG curves were similar. Slight decreases could be observed at the period of temperature between 150 °C and 300 °C, due to the partial decomposition of a-CNT. Since BN did not decompose under 700 °C while CNT did, the char yield of BN@15CNT/PBz composites should theoretically have been lower than that of BN/PBz composites at the same filler loading, theoretically. However, the out-come was completely opposite. Not only was the residual mass of BN@15CNT/PBz composites higher than that of BN/PBz composites at the same filler loading, but also the margin between them increased with the addition of filler loading, which means the existence of CNTs could promote the formation of char residue [43]. In the meanwhile, the T_heat-resistance index_ (°C) of BN@15CNT/PBz and BN/PBz composites revealed that BN@15CNT hybrid fillers had more positive effect on the thermal stability of PBz composites than BN did, which became more obvious with the increase in filler loading. It can be inferred that the introduction of CNTs restricted the thermal motion of tether units, thereby minimizing the number of organic decomposition pathways accessible on the tether, resulting in the improvement of the composites’ thermal properties.

### 3.8. Mechanism of BN@CNT Hybrid Fillers Thermal Conductivity

In general, it is believed that the high thermal boundary resistance (R_B_) between polymer matrix and fillers is the main factor that the experimental λ values derive from theoretical predictions. A lot of theoretical models and simulation models of composites have been proposed in past decades to estimate the R_B_ value. Herein, an effective medium approximation (EMA) was employed to analyze the obtained data in order to understand the effect of CNTs in the BN@15CNT hybrid filler on the improvement of the composites’ thermal conductivity. The mathematical formula of λ value of composites in the EMA model are shown as follows [13]:

(3) $$\lambda {}_{c}=\lambda_{m}\frac{3+2V{}_{f}\left(\frac{\lambda {}_{f}-\lambda {}_{m}}{\lambda {}_{f}}\right)}{3-V{}_{f}\left(1-\alpha -\frac{\lambda {}_{m}}{\lambda {}_{f}}\right)}$$

(4) $$\alpha =\frac{R_{B}\lambda_{m}}{h}$$

in which λ_f_ and λ_m_ stand for the thermal conductivity of the filler and matrix materials, respectively. V_f_ is the volumetric fraction of filler. R_B_ means thermal boundary resistance. h represents thickness of the BN, which was estimated as 300 nm according to SEM observation. The thermally conductive coefficient of BN was 200 W· m^−1^· K^−1^, and that of BN@15CNT can be estimated through the following equation [44]:

(5) $$\lambda =\lambda {}_{1}f+\lambda {}_{2}\left(1-f\right)$$

in which λ, λ_1_ and λ_2_ represent the thermally conductive coefficient of the BN@15CNT hybrid filler, BN and CNTs, respectively. f means the volumetric fraction of BN in the BN@15CNT hybrid filler. The λ value of CNTs is 1300 W· m^−1^· K^−1^. Therefore, the λ value of the BN@15CNT hybrid filler is 290 W· m^−1^· K^−1^.

**Figure 13:**
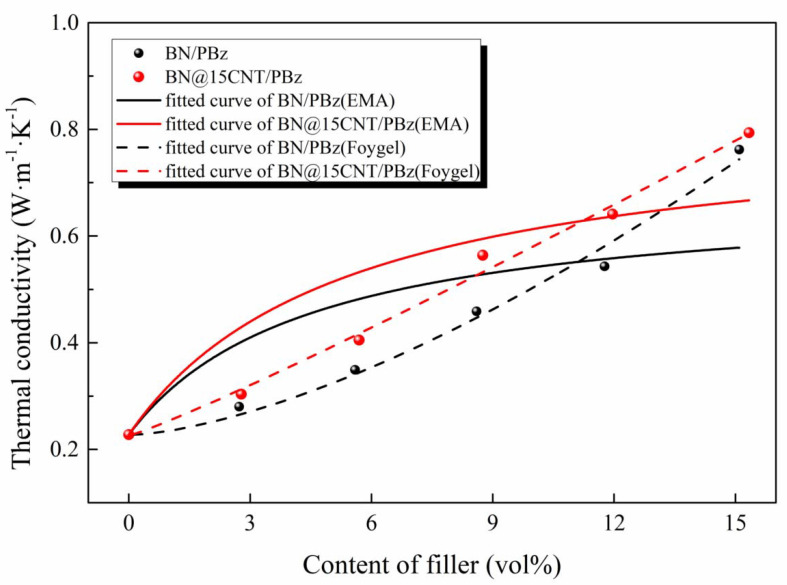
Fitted curves of λ values of BN/PBz and BN@15CNT/PBz composites with the effective medium approximation (EMA) model and Foygel’s model.

Figure 13 and Table 4 show the fitted curves and parameters of the EMA model, with which the two R_B_ values were calculated. The R_B_ value of BN/PBz was 2.194 × 10^−6^ m^2^· K W^−1^, while that of BN@15CNT/PBz was 2.046 × 10^−6^ m^2^· K W^−1^. The slight difference between them indicated the negligible effect of the CNTs on the heat conduction and thermal coupling to the PBz at low filler loading.

The simple EMA model for BN and BN@15CNT is more accurate for the composites in which filler particles are separated by the polymer matrix. Therefore, the λ values will be underestimated when the content of filler is sufficient for the construction of thermally conductive channels. With the increasing in filler loading, the main reason leading to phonon scattering turned into thermal contact resistance generated between filler instead of interfacial thermal resistance generated between filler and polymer matrix [45]. To illustrate the effect of BN@15CNT hybrid filler on enhancing thermal conductivity of PBz composites, the Foygel’s model was employed to compute the contact thermal resistance(R_c_) between filler particles. The mathematical formulas of the λ value of composites in Foygel’s model are shown as follows [46]:

(6) $$\lambda -\lambda_{m}=K(\frac{V_{f}-V_{c}}{1-V_{c}}{)}^{\beta }$$

(7) $$R_{c}=\frac{1}{KdV_{c}^{\beta }}$$

in which λ_f_ and λ_m_ stand for the thermally conductive coefficient values of the filler and matrix materials, respectively. *K* refers to a pre-exponential factor ratio which means the estimated contribution of individual filler networks, *β* stands for a thermal conductivity exponent which reflects on the aspect ratio of the fillers, *V*_c_ refers to the critical volumetric fraction of fillers, and *d* means diameter of fillers which is 50µm according to the supplier.

The fitted results and fitted curve for Foygel’s model are shown in Figure 13 and Table 4. The R_c_ value for BN/PBz was calculated to be 8.090 × 10^7^ K· W^−1^, which was about seven times that of BN@15CNT/PBz, which was 1.216 × 10^7^ K· W^−1^. The tremendous difference between them revealed that the phonon scatting phenomenon was undermined to a certain extent. The possible explanation was that CNTs served as interstitial material to connect adjacent BN platelets [47]. Therefore, the contact area was enlarged so that phonons could transfer throughout the composites with less resistance.

## 4. Conclusions

A novel hybrid filler (BN@CNT) with a hierarchical “line-plane” structure was successfully fabricated through a condensation reaction. A series of hybrid fillers prepared with different mass ratios between BN and CNTs, ranging from 100:5 to 100:20. It has been proven that BN@15CNT hybrid fillers exhibited the best abilities in promoting thermal conductivity of PBz composites compared to the other ones, because with insufficient content of CNTs, the edge of BN platelets cannot be covered with CNTs, which hindered the connection between hybrid fillers. The λ value of PBz composite reached 0.794 W· m^−1^· K^−1^ with 25 wt% BN@15CNT hybrid filler, which can be further improved to 0.865 W· m^−1^· K^−1^ with 15 wt% of BN@15CNT and 10 wt% of BN. Theoretical fitting revealed that CNTs acted as an interstitial material, transferring phonons between BN platelets. The ε and tanδ values of PBz composites increases slightly with the increasing content of BN@15CNT hybrid filler. Moreover, the prepared PBz composites maintain high electrically insulating properties, showing their potential applications in packaging materials and circuit boards. TGA revealed that the BN@15CNT hybrid filler slightly increase thermal stability of the composites and promoted the formation of char residue.

## Figures and Tables

**Table 1:** Formula of BN@xCNT.

	BN (g)	CNT (g)	THF (L)	DIC (g)
BN@5CNT BN@10CNT	10	0.5	0.5	2
	10	1	1	2
BN@15CNT	10	1.5	1.5	2
BN@20CNT	10	2	2	2

**Table 2:** Calculated results of content of CNTs and corresponding data used.

	Weight Loss (%)	Content of CNTs (%)	α (%)
BN@5CNT	3.593	3.052	64.10
BN@10CNT	6.672	6.417	70.58
BN@15CNT	9.429	9.430	72.30
BN@20CNT	10.248	10.325	61.95

**Table 3:** Corresponding characteristic thermal data of TGA curves

Sample Name	Weight Loss Temperature (°C)		T_heat-resistance index_ (°C)
	T_5_	T_30_	
PBZ	394	460	212
10 wt%BN@15CNT/PBZ	362	465	208
10 wt%BN/PBZ	367	462	208
25 wt%BN@15CNT/PBZ	372	495	218
25 wt%BN/PBZ	378	483	216

T_Heat-resistance index_ = 0.49 × (T5 + 0.6 × (T_30_ -T_5_)) T_5_ and T_30_ is corresponding decomposition temperature of 5 wt% and 30 wt% weight loss, respectively.

**Table 4:** Fitted parameters of λ values of BN/PBz and BN@15CNT/PBz composites with the EMA model and Foygel’s model.

	EMA		Foygel			
	α	RB(m^2^· K· W^−1^)	Vc	β	K	Rc(K· W^−1^)
BN/PBz	1.665	2.194 × 10^−6^	0.100	1.510	0.008	8.090 × 10^7^
BN@15CNT/PBz	1.553	2.046 × 10^−6^	0.080	1.093	0.026	1.216 × 10^7^
